# Long-term effects of simulated gastric juice alternated with brushing on hardness, substance loss, flexural strength and reliability of CAD-CAM monolithic materials

**DOI:** 10.1590/1678-7757-2021-0536

**Published:** 2022-04-29

**Authors:** Jailson Rodrigues Oliveira, Marlon Eduardo Menezes da Cruz, Lívia Nordi Dovigo, Renata Garcia Fonseca

**Affiliations:** 1 Universidade Estadual Paulista Faculdade de Odontologia de Araraquara Departamento de Materiais Odontológicos e Prótese Araraquara SP Brasil Universidade Estadual Paulista (UNESP), Faculdade de Odontologia de Araraquara, Departamento de Materiais Odontológicos e Prótese, Araraquara, SP, Brasil.; 2 Universidade Estadual Paulista Faculdade de Odontologia de Araraquara Departamento de Odontologia Social Araraquara SP Brasil Universidade Estadual Paulista (UNESP), Faculdade de Odontologia de Araraquara, Departamento de Odontologia Social, Araraquara, SP, Brasil.

**Keywords:** Computer-aided design, Hydrochloric acid, Toothbrushing, Physical properties, Mechanical tests

## Abstract

**Objectives:**

The purpose of this study is to evaluate, over a simulated 5-year period, the effect of simulated gastric juice alternated with brushing on CAD-CAM monolithic materials considering microhardness, substance loss, flexural strength, and reliability of the materials.

**Methodology:**

Blocks from Lava Ultimate (LU), Vita Enamic (VE), IPS Empress CAD (EMP), IPS e.max CAD (EMAX), and Vita Suprinity (VS) were milled into cylinders and sliced into disks. The EMAX and VS were crystallized, and all specimens were polished with silicon carbide papers and allocated as follows: 1) artificial saliva + brushing or 2) simulated gastric juice (0.113% hydrochloric acid (HCl) solution in deionized water, pH 1.2) + brushing, simulating 1, 3, and 5 years of clinical function. Each year of clinical function was simulated by three repetitions of immersion for 3 hours in artificial saliva or simulated gastric juice followed by 1,217 brushing cycles. The microhardness and substance loss were evaluated at baseline (T0) and at each year by using a Vickers hardness tester and an analytical balance. The biaxial flexural strength (BFS) test was performed in a mechanical testing machine at the end of the 5th year. Weibull modulus was calculated from the BFS data.

**Results:**

The microhardness of the LU was not influenced by the treatment, whereas that of the other materials, in certain years, was significantly lower in the gastric juice + brushing groups in comparison with artificial saliva + brushing groups. In general, the materials did not present a significant change in microhardness over time, for either of the treatments. The LU alone showed greater substance loss in the gastric juice + brushing groups for every year. In both treatments, the LU, VE, and EMP exhibited a significant increase in the substance loss over time. The treatment did not affect the BFS of the materials. The gastric juice + brushing decreased the reliability of the VE.

**Conclusions:**

All materials were somehow impaired by the gastric juice + brushing in at least one of the evaluated parameters, except for the BFS. However, in a deeper analysis, the LU would be the least indicated materials, followed by VE, for patients with eating disorders.

## Introduction

The possibility of fabricating satisfactory restorations of a single-session application, regarding several aspects,^1^ boosted the development of CAD/CAM monolithic materials of varied compositions, such as composite resins (Lava Ultimate, CERASMART, KATANA AVENCIA Block, Grandio blocs, SHOFU Block HC, BRILLIANT Crios), polymer-infiltrated ceramic network (Vita Enamic), feldspathic porcelain (VITABLOCS^®^ Mark II, CEREC Blocs), leucite reinforced glass ceramic (IPS Empress CAD), lithium disilicate (IPS e.max CAD), zirconia-reinforced lithium silicate (Vita Suprinity, Celtra Duo), and different zirconia ceramic generations. Many aspects of these materials, such as microstructure,^2 - 4^ mechanical and optical properties,^2 - 8^ among others, have already been extensively investigated, providing subsidies to use them safely.

However, since they are monolithic, these materials are exposed to the oral environment, under the action not only of extrinsic factors, such as acids from food, but also of intrinsic factors, among which the gastric juice can be highlighted in patients with bulimia nervosa or gastroesophageal reflux, the prevalence of which has been increasing due to bariatric surgeries.^9^ Despite the strong scientific and clinical evidence supporting the adverse effects of the gastric juice on both the tooth structure^10 , 11^ and restorative materials, like direct and indirect composite resins,^12 , 13^ few studies have investigated the impact of this acidic challenge on CAD/CAM monolithic materials.

To the best of our knowledge, nine studies^14 - 22^ were found evaluating the effect of hydrochloric acid on different aspects of CAD/CAM monolithic materials such as roughness,^14 - 20 , 22^ topography,^14 , 16 , 17 , 19 , 22^ hardness,^16 , 19^ substance loss,^14 , 19^ optical properties,^14 , 19 - 22^ and strength.^17 , 20^ Studies evaluating the effect of brushing on these materials were also found,^22 - 27^ two ^26 , 27^ of which were the only ones to evaluate the impact of brushing in association with food-based acids. However, only two studies^20 , 22^ evaluated the effect of gastric juice in association with brushing. In one of them,^20^ the acidic challenge was performed first, followed by abrasion, simulating a two-year period. The evaluation of the effect of gastric juice alternated with brushing would represent more closely what happens clinically in cases of bulimia nervosa, considering that bulimic patients regurgitate on average 3x/day^13 , 28^ and that they usually brush the teeth right after each episode in an attempt to hide the eating disorder.^29 , 30^ Additionally, it is known that the adverse effects of the bulimia nervosa in the oral cavity become more evident two years after the onset of the eating disorder,^31 , 32^ indicating the importance of evaluating longer periods. This scenario was only found in the study by Cruz, et al.^22^ (2020) wherein the effect of the gastric juice alternated with brushing, simulating up to five years of clinical function, was evaluated. However, the authors^22^ focused on the roughness, topography, and staining susceptibility of CAD-CAM monolithic materials. Therefore, studies that evaluate the effect of this same scenario but on other properties are urgently needed.

The purpose of our study is to evaluate, over a simulated five-year period, the effect of simulated gastric juice alternated with brushing on microhardness, substance loss, flexural strength, and reliability of CAD-CAM monolithic materials. The null hypotheses is that the acidic challenge alternated with brushing would not promote statistically different microhardness, substance loss, and biaxial flexural strength when compared to brushing alone.

## Methodology

### Specimen preparation and treatments

**Figure 1:**
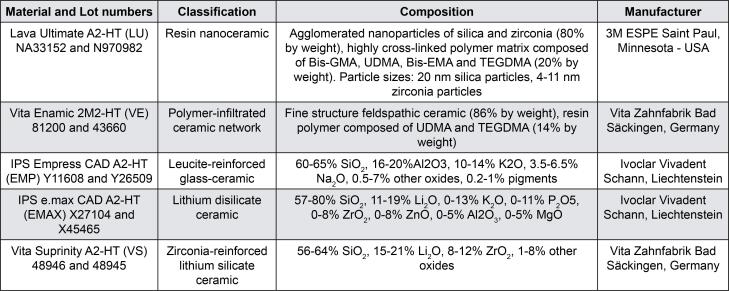
CAD-CAM monolithic materials evaluated

The following materials were tested: Lava Ultimate (LU), Vita Enamic (VE), IPS Empress CAD (EMP), IPS e.max CAD (EMAX), and Vita Suprinity (VS). Figure 1 shows the classification, composition, and manufacturer for each of the evaluated materials. The sample size for the microhardness and substance loss analyses (n=10) was preliminarily defined based on the researchers experience in previous studies with the same response variables. Once the data were obtained, the effect size estimates were used to check the adequacy of the sample size through formal calculations (GPower; F-tests ANOVA: Repeated measures, within-between interaction; α=0.05; β=0.20). Regarding the BFS, since the Weibull modulus was calculated from the BFS data, n=30 was used.

CAD-CAM blocks were milled into cylinders (Ø=10.0 mm) and sliced into disks of approximately 1.3 mm ±0.02 mm with a precision saw (IsoMet 1000; Buehler, Lake Bluff, Illinois, USA), under water irrigation, totalizing 100 disks of each material. The disks had their edges finished with a ceramic polisher (Exa Cerapol 0361HP; Edenta AG, AU/SG, Switzerland) in a low-speed handpiece. The EMAX and VS disks were crystallized (Programat P310; Ivoclar Vivadent AG) according to the manufacturers’instructions. Afterwards, all disks were polished with silicon carbide papers (600, 1200, 1500 grits) in a polisher (Arapol 2V; Arotec Indústria e Comércio, Cotia, São Paulo, Brazil), under water irrigation. To standardize the disk thickness, metal matrices were used with an internal hole of 10.0 mm diameter and 1.2 mm thick, the surfaces of which was equipped with widea to protect them from polishing. After polishing, the thickness were checked with a caliper and disks with a final thickness of 1.2 mm ±٠.٠٢ mm were accepted.

The disks were randomly allocated into two groups: 1) alternation between artificial saliva and brushing (control), and 2) alternation between simulated gastric juice and brushing; simulating 1, 3, and 5 years of clinical function. Tables 1 and 2 show the protocol used to simulate 1 year of clinical function for each treatment. Since bulimic patients generally regurgitate 3x/day^13 , 28^ and each episode lasts 30 s, to simulate 4 months of exposure to gastric juice, it would take 3 h of immersion in the solution. The simulated gastric juice was prepared with 0.113% hydrochloric acid (HCl) solution in deionized water, pH 1.2.^14 , 19^ Specimens were immersed in 1 ml of artificial saliva or simulated gastric juice in flat-bottom microtiter plates. Regarding brushing, 3,650 cycles was considered to represent 1 year,^25^ resulting in 1,217 cycles to simulate 4 months. Brushing was carried out in a brushing machine (MAVTEC Comércio e Serviços, Ribeirão Preto, São Paulo, Brazil), under 2.0 N and 1 Hz, with an Oral B 40 soft bristles toothbrush, and a solution of distilled water and toothpaste (Colgate, Colgate-Palmolive, São Bernardo do Campo, São Paulo, Brazil) in a 1:1 ratio.^27^ The toothbrushes were replaced every 2 h and 40 min, and the toothpaste slurry was prepared for each new brushing cycle. After each 3-hour period of artificial saliva or simulated gastric juice exposure and 1,217 cycles of brushing, the specimens were washed with deionized water using a spray bottle, followed by 2 cycles of 20 min each in an ultrasonic bath with deionized water.

**Table 1:** year of clinical function simulating the absence of bulimia nervosa

Cycles	Simulation	Correspondence
1 cycle of 4 months	4 months of exposure to artificial saliva	3 h of exposure
	4 months of brushing	1,217 cycles
1 cycle of 4 months	4 months of exposure to artificial saliva	3 h of exposure
	4 months of brushing	1,217 cycles
1 cycle of 4 months	4 months of exposure to artificial saliva	3 h of exposure
	4 months of brushing	1,217 cycles

**Table 2:** 1 year of clinical function simulation the presence of bulimia nervosa

Cycles	Simulation	Correspondence
1 cycle of 4 months	4 months of exposure to gastric juice	3 h of exposure
	4 months of brushing	1,217 cycles
1 cycle of 4 months	4 months of exposure to gastric juice	3 h of exposure
	4 months of brushing	1,217 cycles
1 cycle of 4 months	4 months of exposure to gastric juice	3 h of exposure
	4 months of brushing	1,217 cycles

### Microhardness

Microhardness analysis was performed in a hardness tester (Buehler, Lake Bluff, Illinois, USA); 5 indentations were made in each specimen with a 20-N load and a 20-second dwell time. Vickers hardness number (VHN) were calculated according to the equation H=P/2d^2^, in which P is the load in Newton and d is the average of the diagonal values. Twenty disks were obtained from each material, allocating 10 for each treatment; readings at the baseline (T_0_) and at the end of the 1^st^ (T^1^), 3^rd^ (T_3_), and 5^th^ (T_5_) year were made in the same specimens.

### Substance loss

For the substance loss (μg), 20 additional disks were obtained from each material, being 10 for each treatment. The readings at the baseline (T_0_) and at the end of the 1^st^ (T_1_), 3^rd^ (T_3_), and 5^th^ (T_5_) year were made in the same specimens. Before the readings, the specimens were ultrasonically cleaned in distilled water for 5 minutes and stored in an oven at 37°C for 7 days. For the weighing, the specimens were positioned on an analytical balance (XS105 Dual Range; Mettler Toledo GmbH, Greifensee, Switzerland), and the measurements were recorded after 30 seconds. The difference in mass between each year and the baseline was calculated, resulting in ΔT_0_-T_1_, ΔT_0_-T_3_, and ΔT_0_-T_5._

### Biaxial flexural strength

For the biaxial flexural strength (BFS) test, 60 disks were obtained from each material – 30 for each treatment. This analysis was performed after the 5^th^ year of each treatment in a mechanical testing machine (model DL2000, EMIC Equipment and Systems Testing, São José dos Pinhais, Paraná, Brazil), according to the ISO 6872 standard.^33^ The disks were placed centrally over three stainless steel balls (2.5 mm diameter) positioned 120° apart on a support circle (8.0 mm diameter). A flat punch running at a crosshead speed of 0.5 mm/min directed a uniaxial tensile load to the treated surfaces until failure. The BFS was calculated using the following equation:


$$\text{S}=-0.2387\;\text{P}\left(\text{X}-\text{Y}\right)/{\text{d}}^{2}$$

in which S is the biaxial flexural strength (MPa), P is the fracture load (N), and d is the disk specimen thickness at the fracture site (mm). X and Y were determined as follows:


$$\begin{array}{l} \text{X}=\left(1+\text{v}\right)\;\ln{\left(\text{r}2/\text{r}3\right)}^{2}+\left[\left(1-\text{v}\right)/2\right]{\left(\text{r}2/\text{r}3\right)}^{2}\text{and}\;\\ \text{Y}=\left(1+\text{v}\right)\;[1+\ln{\left(\text{r}1/\text{r}3\right)}^{2}]+\left(1-\text{v}\right){\left(\text{r}1/\text{r}3\right)}^{2} \end{array}$$

in which v is the Poisson’s ratio (0.25), r1 is the radius of support circle, r2 is the radius of loaded area, and r3 is the radius of the specimen.

### Statistical Analysis

Microhardness data and differences in mass (ΔT_0_-T_1_, ΔT_0_-T_3_, and ΔT_0_-T_5_) were submitted to mixed repeated-measures ANOVA with time as the within-subjects factor, and material and treatment as the between-subjects factors. Normality and sphericity were verified by Shapiro-Wilk (microhardness: *P≥* 0.066; mass: *P≥* 0.047) and Mauchly (microhardness: *P* =0.111; mass: *P* <0.001) tests. Since the assumption of Mauchly’s sphericity for the differences in mass was not met, the lower limit was used to calculate the Epsilon correction factor (ε=0.5). The Bonferroni test was used to assess differences among groups. The BFS data were submitted to 2-way ANOVA with material and treatment as independent variables. Normality and homoscedasticity were verified by Shapiro-Wilk ( *P>* 0.178) and Levene ( *P* <0.001) tests. Although the assumption of homoscedasticity was not met, we decided to proceed with the ANOVA, which is known to be robust for moderate deviations from normality and homoscedasticity in cases of block design with balanced groups,^34^ and the Games-Howell test was employed to assess the differences among groups. Weibull modulus was calculated from the BFS data. The level of significance was set at 0.05. The statistical analyses were performed using the IBM SPSS Statistics v22.0 statistical software.

## Results

For microhardness, the isolated variables (P<.001) and the interactions material*treatment ( *P* <.01), treatment*time ( *P* <.001), and material*treatment*time ( *P* <.001) were significant, except for material*time ( *P* =.150). The effect size (η^2^_p_) of the isolated variables material, treatment, and time was respectively 1.000, 0.683, and 0.180. Table 3 shows the microhardness mean values, standard deviations, and statistical comparisons. The LU was not influenced by the treatment; whereas the other materials were, with significant lower microhardness in the gastric juice + brushing groups, in certain years. In general, for both treatments, the materials did not present a significant change in microhardness over the years. Regardless of the treatment and time, the order of microhardness of the materials was: VS>EMAX>EMP>VE>LU.

**Table 3:** Mean microhardness mean values (VHN) ± SD, and statistical comparisons

		T0	T1	T3	T5
LU	saliva + brushing	97.7 ±2.8^Af^	102.7 ±3.7^Ah^	109.2 ±2.9^Ag^	108.6 ±4.8^Ai^
	gastric juice + brushing	100.2 ±1.5^Af^	96.2 ±1.9^Ah^	99.4 ±2.1^Ag^	100.5 ±1.8^Ai^
VE	saliva + brushing	207.2 ±8.8^Ae^	207.7 ±7.0^Ag^	220.0 ±17.1^Ae^	219.0 ±7.5^Ag^
	gastric juice + brushing	198.2 ±6.0^Ae^	203.2 ±6.6^Ag^	197.8±7.5^Af^	202.0 ±7.2^Ah^
EMP	saliva + brushing	470.5 ±15.8^Ad^	481.4 ±7.8^Ae^	479.9 ±7.7^Ac^	483.5 ±9.3^Ae^
	gastric juice + brushing	458.7 ±6.0^Ad^	465.3 ±10.3^Af^	464.8 ±6.2^Ad^	462.6 ±6.9^Af^
EMAX	saliva + brushing	507.4 ±8.9^Bc^	523.6 ±9.1^Ac^	514.1 ±3.9^ABb^	527.0 ±4.2^Ac^
	gastric juice + brushing	511.3 ±9.6^Ac^	501.0 ±8.8^Ad^	509.3 ±7.1^Ab^	510.8 ±4.3^Ad^
VS	saliva + brushing	577.7 ±8.1^Aa^	581.0 ±15.9^Aa^	584.1 ±9.2^Aa^	587.7 ±5.5^Aa^
	gastric juice + brushing	562.6 ±6.3^Ab^	563.0 ±7.6^Ab^	574.2 ±6.5^Aa^	566.8 ±7.9^Ab^

Different lowercase letters indicate significant difference in columns (P<.05).

Different uppercase letters indicate significant difference in lines (P<.05).

For the substance loss, the isolated variables (P<.001) and the interactions material*treatment ( *P* <.001), material*time ( *P* <.001), and material*treatment*time ( *P* =.003) were significant. The effect size (η^2^_p_) of the isolated variables material, treatment, and time was respectively 0.936, 0.224, and 0.829. Table 4 shows the Δ mass mean values, standard deviations, and statistical comparisons. Only the LU, showed greater substance loss in the gastric juice + brushing groups, in all years. The LU, VE, and EMP, in both treatments, and the VS in the artificial saliva + brushing, exhibited a significant increase in substance loss over the years. In general the LU showed the greatest substance loss, followed by the VE, for both treatments and in each of the three years evaluated; whereas there was no significant difference among the EMP, EMAX, and VS.

**Table 4:** Δ mass mean values (μg) ± SD, and statistical comparisons

		ΔT0 –T1	ΔT0 –T3	ΔT0 –T5
LU	saliva + brushing	468.3±36.1^Bb^	408.7±113.1^Bb^	804.3±197.3^Ab^
	gastric juice + brushing	595.0±43.7^Ba^	621.7±69.0^Ba^	978.7±124.9^Aa^
VE	saliva + brushing	157.7±44.0^Bcd^	338.3±82.0^Ab^	411.6±96.4^Acd^
	gastric juice + brushing	237.0±34.9^Bc^	383.2±48.4^Ab^	489.7±87.8^Ac^
EMP	saliva + brushing	48.3±24.0^Bde^	137.1±39.8^ABc^	233.7±70.8^Aef^
	gastric juice + brushing	69.9±12.4^Bde^	177.3±51.7^Bc^	305.4±94.5^Ade^
EMAX	saliva + brushing	22.0±22.5^Ae^	89.3±37.3^Ac^	133.7±33.5^Af^
	gastric juice + brushing	39.5±50.8^Ade^	108.7±40.0^Ac^	148.2±74.7^Af^
VS	saliva + brushing	4.3±36.8^Be^	87.0±59.7^ABc^	149.7±100.3^Af^
	gastric juice + brushing	10.5 ±30.3^Ae^	100.1 ±31.7^Ac^	128.0 ±59.8^Af^

Different lowercase letters indicate significant difference in columns (P<.05).

Different uppercase letters indicate significant difference in lines (P<.05).

**Figure 2:**
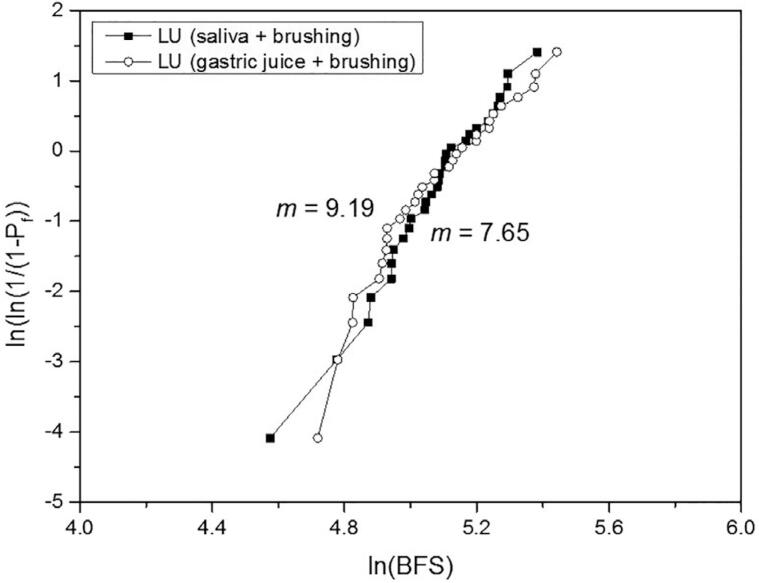
Weibull graph of both treatments for the LU

**Figure 3:**
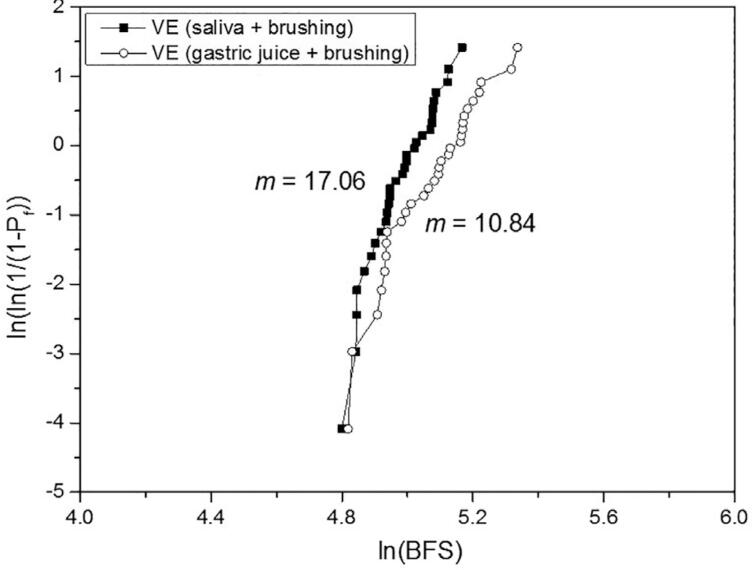
Weibull graph of both treatments for the VE

**Figure 4:**
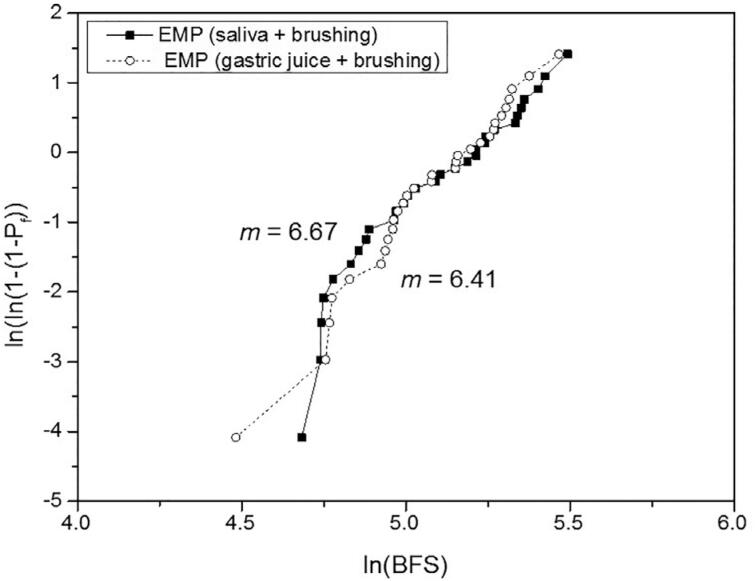
Weibull graph of both treatments for the EMP

**Figure 5:**
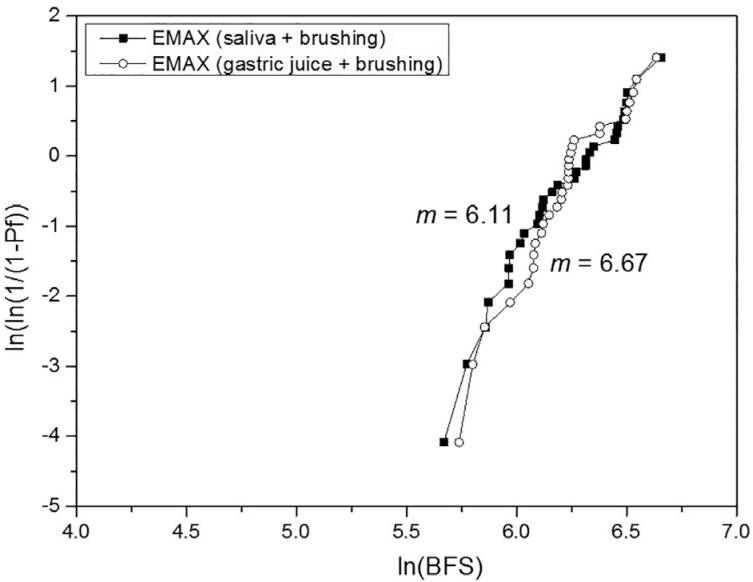
Weibull graph of both treatments for the EMAX

**Figure 6:**
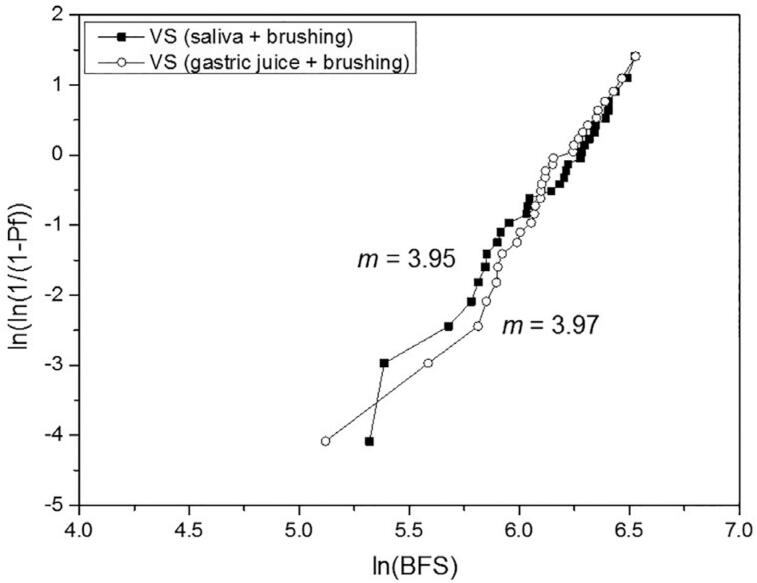
Weibull graph of both treatments for the VS

For the biaxial flexural strength, the 2-way ANOVA indicated significance only for the material (P<.001), whereas the treatment (P=.841) and the interaction material*treatment (P=.969) were not significant. The effect size (η^2^_p_) of the isolated variables material and treatment was respectively 1.000 and 0.069. Table 5 shows the BFS mean values, standard deviations, statistical comparisons, and Weibull modulus. The treatment did not affect the BFS of the materials, which presented the following order: (VS=EMAX) > (EMP=LU=VE). Differently from the other materials, the reliability of the VE was substantially reduced by the gastric juice + brushing when compared to the artificial saliva + brushing. Weibull graphs are presented in Figures 2 , 3 , 4 , 5 , and 6 .

**Table 5:** BFS mean values (MPa) ± SD, statistical comparisons and Weibull modulus (m)

	Lava Ultimate	Vita Enamic	IPS Empress CAD	IPS e.max CAD	Vita Suprinity
saliva + brushing	162.3±26.5^Ab^	146.8±14.1^Ab^	165.9±38.8^Ab^	513.0±127.0^Aa^	466.0±127.5^Aa^
(m)	7.65	17.06	6.67	6.11	3.95
gastric juice + brushing	163.4±31.0^Ab^	161.5±21.6^Ab^	163.6±34.7^Ab^	513.2±111.2^Aa^	461.4±112.4^Aa^
(m)	9.19	10.84	6.41	6.67	3.97

Different uppercase letters indicate significant difference in columns (P<.05).

Different lowercase letters indicate significant difference in lines (P<.05).

## Discussion

The aim of our study was to compare the effects of the gastric juice with artificial saliva when alternated with brushing on CAD-CAM monolithic materials in terms of microhardness, substance loss, BFS, and reliability.

Our first null hypothesis was rejected considering that the microhardness for most of the materials was significantly lower in the gastric juice + brushing groups in certain years, except for the LU. To the best of our knowledge, there are no studies that have evaluated the effect of the gastric juice alternated or associated with brushing on CAD-CAM monolithic materials considering either microhardness or substance loss. However, some related studies, such as that by Cruz, et al.^19^ (2020), observed that the immersion in simulated gastric juice for 24 h did not affect the hardness of the LU, VE, EMAX, and VS. Backer, et al.^16^ (2017) reported the same behavior after the LU was immersed in a similar solution for 6 and 24 h. These studies^16 , 19^ showed that the gastric juice per se had no potential to affect the hardness of the evaluated materials, at least until 24 h of immersion. Even so, in the present study, considering the synergistic effect of brushing, the LU did not show significant differences between either treatment, which at first glance might be attributed to its high polymerization degree and density of the cross-links. Regarding the glass-ceramic materials, Cruz, et al.^19^ (2020) found no significant effect of the simulated gastric juice on their microhardness; whereas other studies^14 , 15 , 18^ that evaluated immersion periods longer than 24 h in other properties, showed that, to a greater or lesser extent, the EMP and EMAX suffered the effects of the same acidic challenge, resulting the dissolution of the leucite and lithium disilicate crystals^15^ or of both the crystalline phase and the vitreous matrix.^14^ Such dissolution was related to the breakdown of the Si-O-Si bonds,^35 , 36^ which could release crystals,^35^ possibly impacting the hardness of the material. Given the above, it can be assumed that the gastric juice alternated with brushing anticipated the degradation process of the glass-ceramics, considering the decrease in their microhardness already in the 1^st^ year. Alencar-Silva, et al.^26^ (2019) reported a significant decrease in hardness after the EMAX was immersed in acidic beverages followed by brushing for a simulated 5-year period. As for the VE, being a porous sintered feldspathic ceramic network infiltrated with polymer, it was subject to all processes previously described. Therefore, it is difficult to estimate how much each phase contributed to the decrease in its microhardness observed in the gastric juice + brushing groups in certain years. The microhardness relationship among the materials did not change with the treatment and time, which can be corroborated by some studies.^5 , 19 , 23 , 27^ Certainly, such differences among the materials are related to the differences in their composition and microstructure.^27^ All these findings are in line with the analysis of the effect size that showed that the material, followed by the treatment it received, had greater impact on microhardness than the time.

The substance loss analysis showed that the treatment affected only the LU, which exhibited significantly greater substance loss in the gastric juice + brushing groups for all years, leading to the rejection of the second null hypothesis. We found only two studies^14 , 19^ that evaluated this aspect after immersion of CAD-CAM monolithic materials in hydrochloric acid. Sulaiman, et al.^14^ (2015) reported a substance loss of 3.05% for the EMAX after a 96-hour immersion period, while Cruz, et al.^19^ (2020) reported no substance loss after the LU, VE, EMAX, and VS were immersed for 24 h. In our study, the substance loss observed in the LU was possibly due to the synergic effect of brushing. In a parallel study,^22^ in which the effect of the same challenges was investigated (but on other properties, including topography), it was observed that the polymeric and inorganic phases of the LU were affected by both treatments; with the difference that, while the artificial saliva + brushing provided a gradual increase in the texture over the years – which is in agreement with the study by Mühlemann et al.^25^ – the gastric juice + brushing seems to have progressively smoothed the surface. Considering that the LU contains Bis-GMA and that this monomer may present a softening by simulated gastric juice,^12 , 13 , 19 , 37 , 38^ it could be assumed that the smoothness was a result of the removal of the disaggregated material by brushing, which would justify the greater substance loss found for the LU in the gastric juice + brushing groups and would lead us to question whether this process would not have been the real reason for the lack of significance between both treatments in the microhardness of the LU. On the other hand, the substance loss of the other materials was not significantly influenced by the treatment. This finding is in line with the study by Cruz, et al.^22^ (2020), in which there were no noticeable topographic changes when the EMP, EMAX, and VS were submitted to either treatment. On the other hand, the more evident topographic changes revealed by the SEM images for the VE in the gastric juice + brushing groups^22^ did not impact its substance loss in our study. The increase in the substance loss observed over the years for the LU, VE, and EMP, in both treatments, and the VS in the artificial saliva + brushing, indicates that brushing, per se, can cause a progressive loss of elements in these materials, as observed by Andrade, et al.^24^ (2020) for the VE. The topographic images taken by Cruz, et al.^22^ (2020) can help us understand the high substance loss exhibited by the LU and VE, over time, for both treatments – with few exceptions. This reflects the higher vulnerability of the polymer phase in losing substance when compared to the ceramic phase. Although no other study has been found for comparison, our study found that the type of treatment was the least decisive factor in determining substance loss, whereas time and material had a very high effect on this parameter.

The third null hypothesis was accepted, since there was no significant difference between the treatments. This finding can be corroborated by Kulkarni, et al.^20^ (2020) who did not observe a significant difference in the BFS when the EMAX was only brushed or exposed to hydrochloric acid and subsequently brushed. No other study with a similar protocol was found for comparison. In our study, the BFS was strongly determined by the material, as revealed by the effect size analysis. The higher strength of the glass-ceramics EMAX and VS in comparison with the other materials evaluated in this study is already known.^2 - 4 , 6^ On the other hand, previous studies,^2 , 4 , 5^ in which no treatment was done, reported higher strength for the VS or for the similar material Celtra Duo in relation to the EMAX, attributing this superiority to the presence of zirconia particles. It is possible that the treatments applied in our study may have affected the BFS of the VS. Regarding their Weibull modulus, which showed no difference between the treatments, that of the VS was 35 to 41% lower than that of the EMAX. In the study by Sen and Us,^4^ the Weibull modulus of the VS was 18% lower; and in the study by Elsaka and Elnaghy^5^ (2016), it was higher than that of the EMAX. The lower performance of the VS, when compared to the EMAX, indicates the need to investigate the VS from different perspectives and under adverse conditions, in order to have a broader understanding of this material. The statistical similarity of BFS between the VE and EMP can be corroborated by other studies.^3 , 6^ However, the Weibull modulus of the VE remained higher than that of the EMP (which did not vary according to the treatment), even after showing a considerable reduction with the gastric juice + brushing treatment, which may be related to the degradation of both phases.^22^ The higher Weibull modulus of the VE in relation to that of the EMP, EMAX, and VS was also reported by Stawarczyk, et al.^6^ (2015); they related it to its lower elastic modulus, which reduces the probability of spontaneous fracture. On the other hand, the statistical similarity of the LU in relation to the VE and EMP can be corroborated by some studies,^2 , 5 , 7^ whereas others^3 , 4 , 6 , 8 , 17^ reported higher strength for the LU. Possibly, the damage suffered by the LU in both treatments,^22^ which resulted in the highest substance loss, may have equated its strength with that of the VE and EMP. Egilmez, et al.^17^ (2018) reported that the LU exhibited greater strength than the VE, even after immersion in hydrochloric acid; in their study, however, brushing was not performed, which could change the strength relationship between these two materials. The higher reliability of the LU in relation to the ceramic materials, as well as its lower reliability in relation to the VE can be corroborated by Stawarczyk, et al.^6^ (2015). The higher Weibull modulus of the VE when compared to that of the LU are also in line with previous studies.^4 , 8^ According to Lim, et al.^8^ (2016) this is due to the presence of the polymeric phase within the ceramic structure that gives plasticity under load, increasing the crack resistance.

After making a deeper analysis based on the results of our study and those found in the literature, we believe that, despite all materials showing a higher tendency to degradation in gastric juice+brushing, the ceramic would be the most suitable for patients with eating disorders. Future studies that evaluate other properties of the CAD-CAM monolithic materials under the same scenario should be conducted to reinforce or challenge our finding. Some limitations of our study include the non-performance of the SEM analysis on the fractured specimens to reveal differences in failure types among the experimental groups and the impossibility of simulating exactly the scenario that occurs clinically, which might influence the materials behavior.

## Conclusion

Considering the results of this *in vitro* study, it was possible to conclude that all materials were somehow impaired by the gastric juice+brushing in at least one of the evaluated parameters, except for the BFS. All materials, except for the LU, showed a decrease in their microhardness in the gastric juice+brushing groups in comparison with artificial saliva+brushing groups, in certain years ( *P* ≤0.019). However, for both treatments, the materials did not present a significant change in microhardness over the years. In all years, the LU showed greater substance loss in the gastric juice+brushing groups ( *P* ≤0.049). In both treatments, the LU, VE, and EMP, exhibited a significant increase in the substance loss over the years. ( *P* ≤0.042). The treatment did not affect the BFS of the materials. Finally, the gastric juice+brushing decreased the reliability of the VE. In a deeper analysis, the LU would be the least indicated materials, followed by VE, for patients with eating disorders.
